# Supplementary material for: The role of side-branching in microstructure development in laser powder-bed fusion
Source: Nat Commun. 2020 Feb 6;11:749. doi: 10.1038/s41467-020-14453-3 (PMC7004990; doi:10.1038/s41467-020-14453-3)
Supplement: Supplementary file 1 — Supplementary information [file 41467_2020_14453_MOESM1_ESM.pdf]

Supplementary information

The role of side-branching in microstructure development in laser powder-bed fusion

Pham *et. al.*

Supplementary Table 1. Material compositions (in wt.%)

	Fe	Cr	Ni	Mn	Co	Mo	C	S	N	P
**316L**	bal.	17.5-18.0	12.5-13.0	2	0.09	2.25-2.5	0.03	0.01	0.06	0.025
**HEA**	bal.	18.2	21.2	17.9	23.2	--	--	--	--	--

Supplementary Table 2: Thermal conductivity and specific heat capacity [1, 2]

Temperature (°C)	Conductivity (W/(mK)		Specific heat capacity (J/K/kg)
	Powder	Bulk	
20	0.1412	14.12	492
100	0.1526	15.26	502
200	0.1669	16.69	514
300	0.1811	18.11	526
400	0.1954	19.54	538
500	0.2096	20.96	550
600	0.2238	22.38	562
700	0.2381	23.81	575
800	0.2523	25.23	587
900	0.2666	26.66	599
1000	0.2808	28.08	611
1100	0.295	29.5	623
1200	0.3093	30.93	635
1300	0.3235	32.35	647
1400	32.78	32.78	659
1500	32.78	32.78	659

Supplementary Table 3: Identified values for parameters used in FEA simulation

Parameter (unit)		Value	Reference
Energy input rate (W)		180	
Energy absorption coefficient		0.6	[3]
Latent heat (kJ/kg)		275	[4]
Convection coefficient W/(m^2K)		5.7	[5]
Liquidus temperature (°C)		1442	
Solidus temperature (°C)		1325	
a_f, a_r, b and c (μm)		35, 135, 120 and 35	Eqn. 2
q_i (kW/m^{-3})		1100	Eqn. 2 [1]
Density (kg/m^{-3})	Powder	4699.9	[6]
	Bulk	7966	

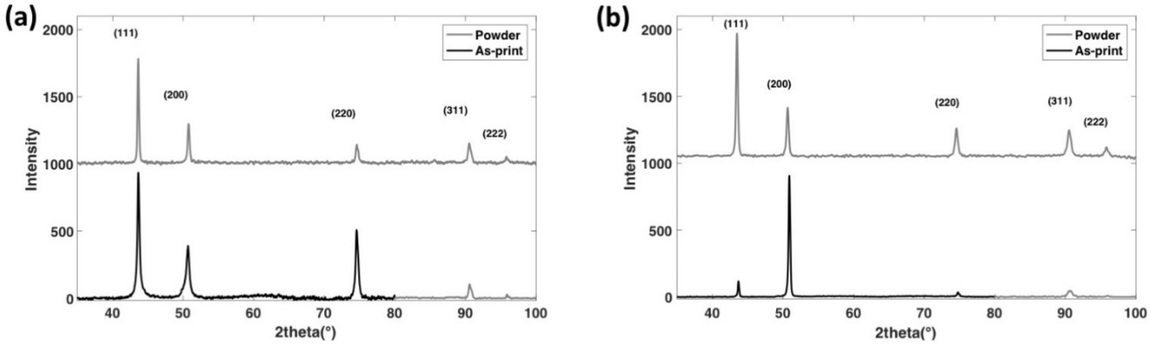

Supplementary Figure 1: X-ray diffraction on sections perpendicular to the build direction. Note: both 316L and HEA used for X-ray diffraction were fabricated by a Renishaw with a bidirectional strategy without rotation between layers. (a) 316L steel. (b) CrMnFeCoNi HEA. The X-ray diffraction confirms they both have single-phase FCC structure in the as-received powder and in builds fabricated by LPBF.

The diffraction profiles of the 316L and HEA are consistent with the pole figures obtained from EBSD scans of the Renishaw 316L (Main Text Figure 5a) and HEA (Main Text Figure 7a). In particular, Main Text Figure 5a and b show that most grains have the <001> inclined to the BD, explaining why the (001) peak observed on the XRD on the section (perpendicular to BD) in Supplementary Figure 1a was not strong. The profiles of the two alloys highlight some minor differences in the crystal orientations on the sections perpendicular to the build direction. While the (200) peak was strongest in the HEA which is consistent with a strong (100) peak closely parallel to the build direction, (220) and (111) were stronger than (200) in 316L, indicating the continuous epitaxial growth was less active and side-branching was more dominant in 316L.

Supplementary Note 1: Finite element model validation and simulation

G and v_i were calculated along the liquidus line and within a steady state melt pool (Main Text Figure 2a-d). In addition, a melt pool was defined as a region bounded within the liquidus temperature isotherm (marked by the inner dashed line in Main Text Figure 2b and c) assuming the nominal composition of the 316L steel. The FEA model identification was done by matching the melt pool dimensions predicted by FEA against the measurement (Main Text Figure 2c versus Supplementary Figure 2). The width and depth of a melt pool simulated by FEA were 147 μm and 107 μm, respectively. These dimensions accurately match the experimentally measured ones that were 145±30 μm in width and 90±20 μm in depth.

In addition, using $\lambda_c = 80{v_i}^{-0.33}G^{-0.33}$ (which is widely believed to represent the relationship between the cooling rate and the cell spacing[7, 8, 9, 10]) and the measured average spacing of cells in 316L (Main Text Figure 2e) gives an experimental measurement of cooling rate in the range of 10^6 K/s while the maximum cooling rate predicted by FEA simulation (Main Text Figure 2) is in the range of 10^5 to 10^6 K/s, suggesting the FEA simulation was reasonably accurate. It can be seen that the angle, θ, between the laser direction and the liquidus isotherm growth direction varies along the solidification front from θ=0 at the surface to θ=90° at the bottom of the melt pool, correspondingly v_i decreases from the laser scan speed, 0.63 m/s^{-1} to close to zero (dotted dash line, Main Text Figure 2d). In contrast to the decreasing trend of v_i, the thermal gradient is a minimum at the top surface and a maximum at the bottom of the melt pool, varying from ~2x10^5 K/m to ~2x10^7 K/m (Main Text Figure 2d) in good agreement with experimental validation that reported the thermal gradients were in the range from 4x10^5 K/m to 2x10^7 K/m [11], further confirming that the reasonable accuracy of the FEA simulation.

The magnitude of the thermal field would be different for the HEA. However, 316L and HEA share multiple similarities in solidification behaviour to other alloys in the Fe-Ni-Cr system [12]. Therefore, the knowledge of thermal profile and solidification obtained from the simulation of 316L steel can be applicable to the HEA.

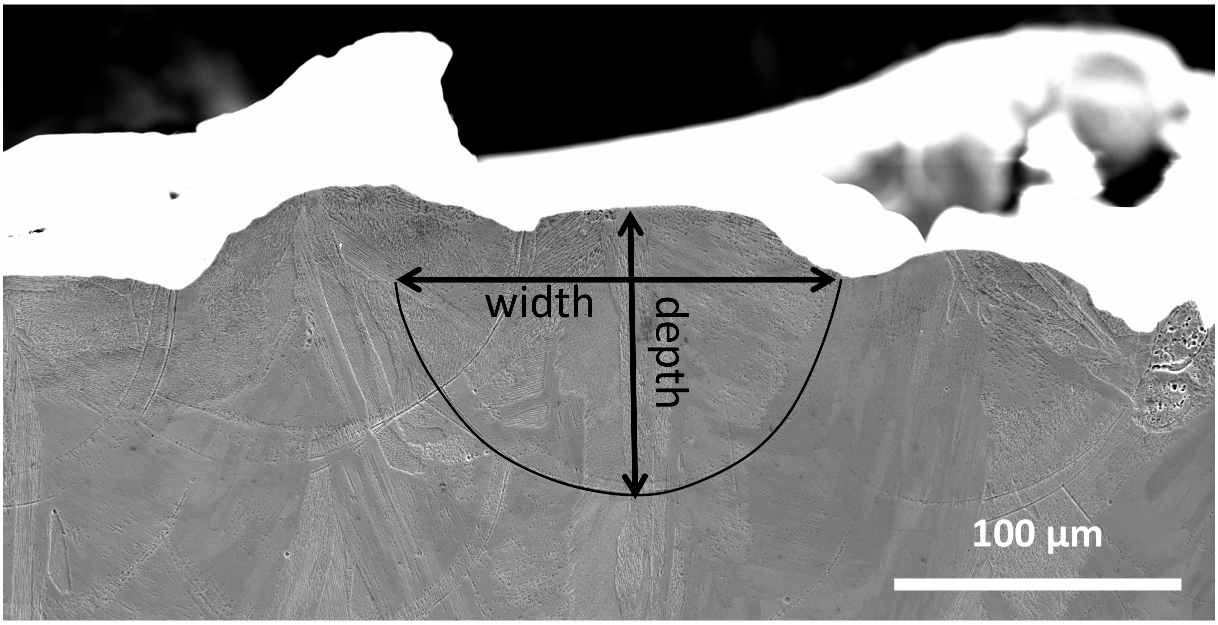

Supplementary Figure 2: Melt pools on the top layer of a 316L build fabricated by Renishaw. The measured dimensions are 90±20 µm in depth and 145±30 µm in width.

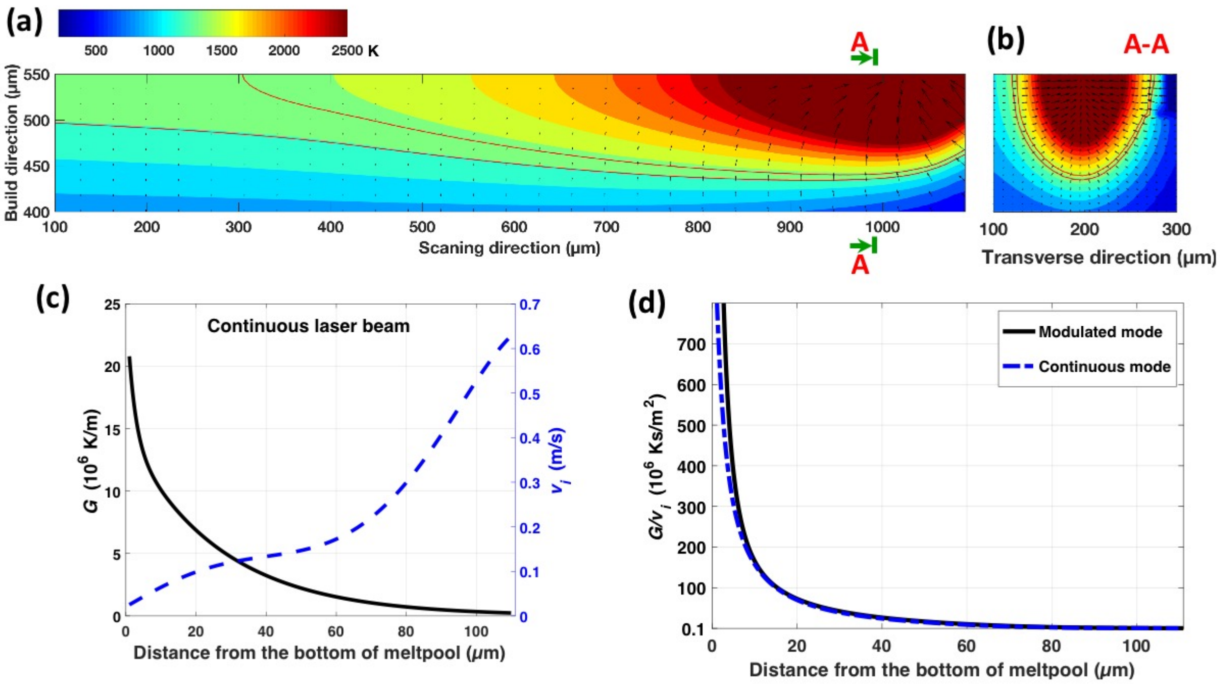

Supplementary Figure 3: Thermal profile associated with a continuous laser beam with the same initial and boundary conditions as those for the Main Text Figure 2. (a) Side-view (parallel to a deposition track) of melt pool: the inner and outer dashed lines are the liquidus and solidus temperatures of the current melt pool, respectively). (b) A-A section transverse to a deposition track (note: kinks of isotherms on the right of melt pool were due to local changes in heat conduction intentionally introduce to mimic the presence of unmelted powder). (c) The variations of G (solid line) and v_i (dotted dash line) along the liquidus front from the bottom towards the top of a melt pool. (d) Variations of the G/v_i from the bottom of a melt pool towards the top of modulated laser mode in comparison to that of the continuous mode.

Supplementary Note 2: Cell spacing versus cooling rates

Because pores can serve as thermal insulation, heat is held longer, leading to reductions in both G and v_i in the regions above pores. According to Eqn. 1, lowering G and v_i leads to coarser cells. However, the decrease in G and v_i depends on the size of pores. The bigger the pore, the larger the reduction. Entrapped gases in laser fusion are usually small. For example, the pore in Main Text Figure 3b was about 20μm. Thereby, the reduction in cooling rate because of entrapped gas pores did not significantly alter the surrounding solidification microstructure. In contrast, large lack-of-fusion pores (e.g. the irregular pore with one dimension larger than 150μm shown in Main Text Figure 3c) cause substantial reduction in G and v_i in the region above the lack-of-fusion pores, resulting in coarser cells above the pore. Following the fitting given in Main Text Figure 2e, $35(v_i G)^{-0.33}$ was used to estimate the change in cooling rate ($|\dot{T}|$) due to the presence of the pore. Assuming that the spacing (λ_1) of microstructure in the region right above the pore in Main Text Figure 3c follows the same relationship, $\lambda_1 = 35(v_i G)^{-0.33} = a(|\dot{T}|)^{-0.5}$. Therefore,

$$\frac{\lambda_c}{\lambda_1} \approx \left(\frac{|\dot{T}_c|}{|\dot{T}_1|}\right)^{-0.33} \qquad \text{Eqn. 1}$$

with $|\dot{T}_c|$ and $|\dot{T}_1|$the cooling rates in the fully consolidated region and in the region right above the large pore in Main Text Figure 3c, respectively. λ_1 of the region above the pore was about 2.3 μm. An estimate of the average cooling rate $|\dot{T}_c|$ is of 1.0x10^6 K/s (Section III.). Thereby, $|\dot{T}_1| = |\dot{T}_c|\left(\frac{\lambda_c}{\lambda_1}\right)^3 \approx 1.0\times10^6\left(\frac{0.63}{2.3}\right)^3 \approx 2.1\times10^4$ K/s. This suggests that the large lack-of-fusion pore in Main Text Figure 3c led to approximately two orders of magnitude reduction in local cooling rate.

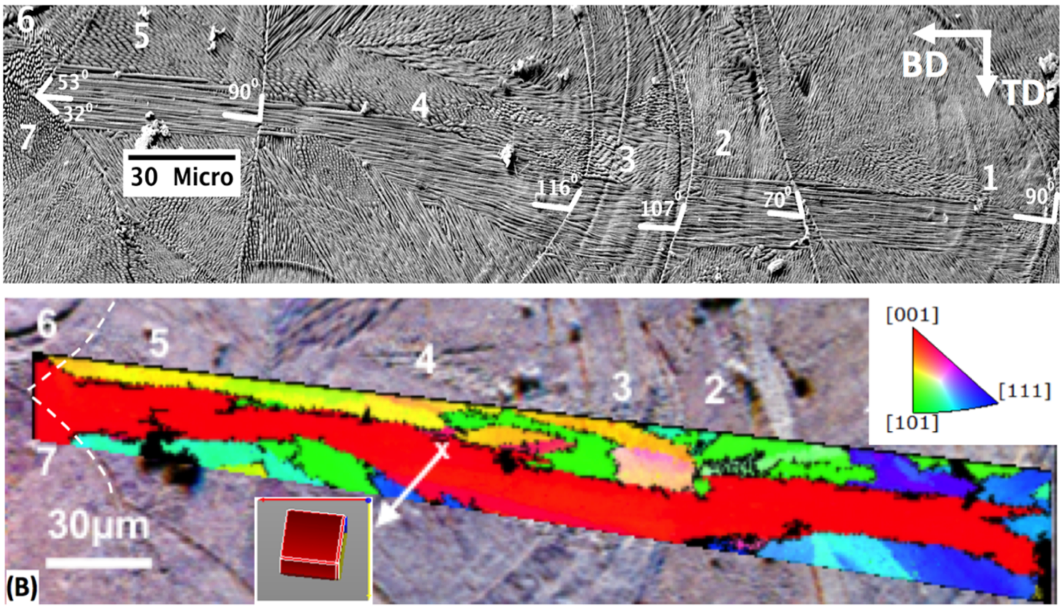

Supplementary Figure 4: Continuous growth of microstructure in 316L fabricated by a Renishaw AM250 with a linear bi-directional scan and 67° of rotation for subsequent layers. (a) An array of cells epitaxially grew across multiple melt pools, (b) the corresponding IPF-BD map. Note: BD stands for build direction while TD is for transverse direction, the dashed line represents the melt-pool boundary. This figure is reused from [13] with permission provided by AIP Publishing.

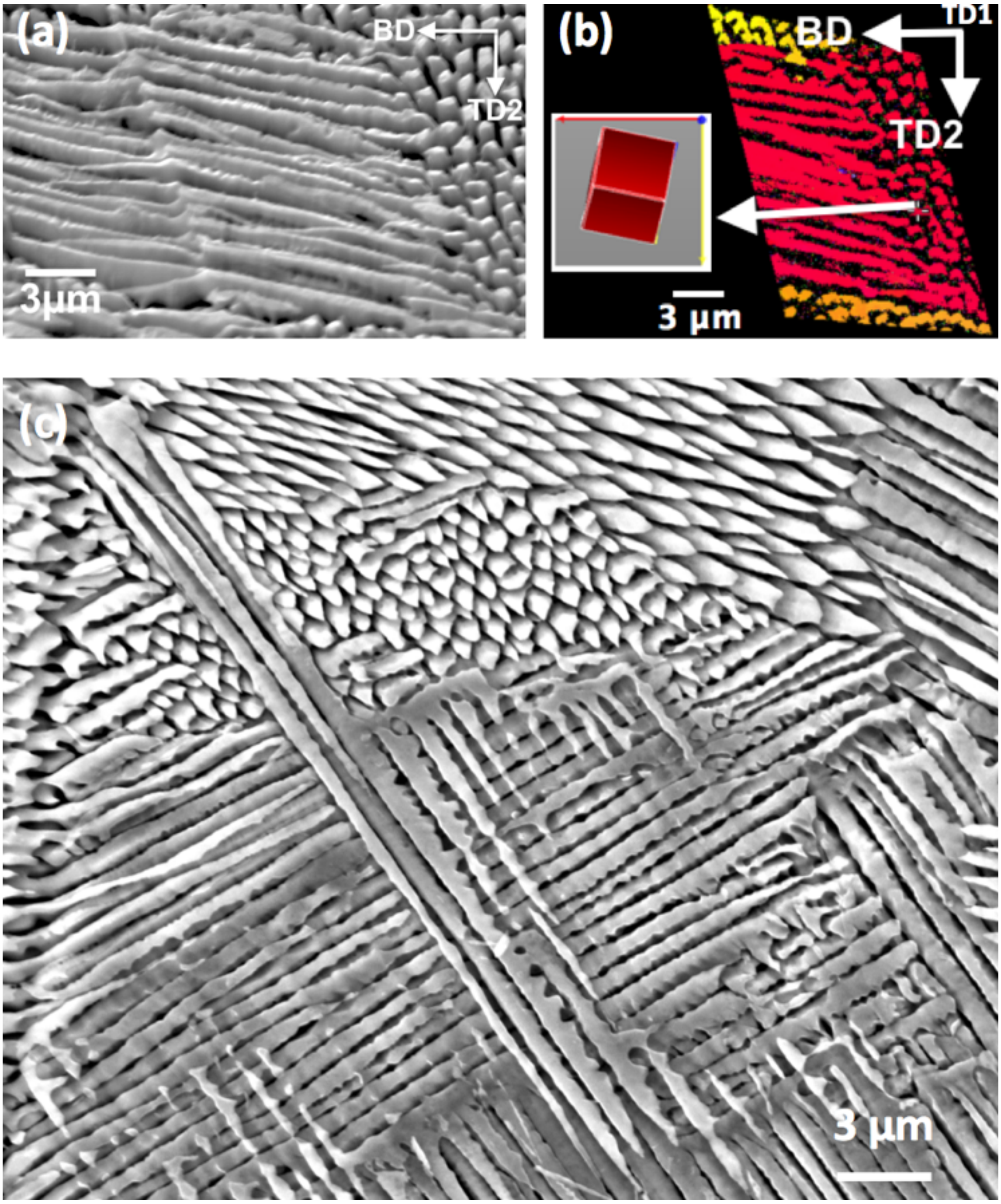

Supplementary Figure 5: Side-branch occurred within a melt pool observed for 316L fabricated with the bidirectional scanning and 67° rotation. (a) SEM image and (b) corresponding IPF along TD1 (which is out of plane). (c) layer-like structure of cells.

Supplementary Figure 5a shows side-branching in the middle of a melt pool: a new in-plane cell domain branched out from the parent cells (which were out of plane in the top right region). and had a growth direction almost perpendicular to the parents, and maintained the parent cell's crystallographic orientation (Supplementary Figure 5b). Supplementary Figure 5c presents a crisscross structure of cells in a melt pool.

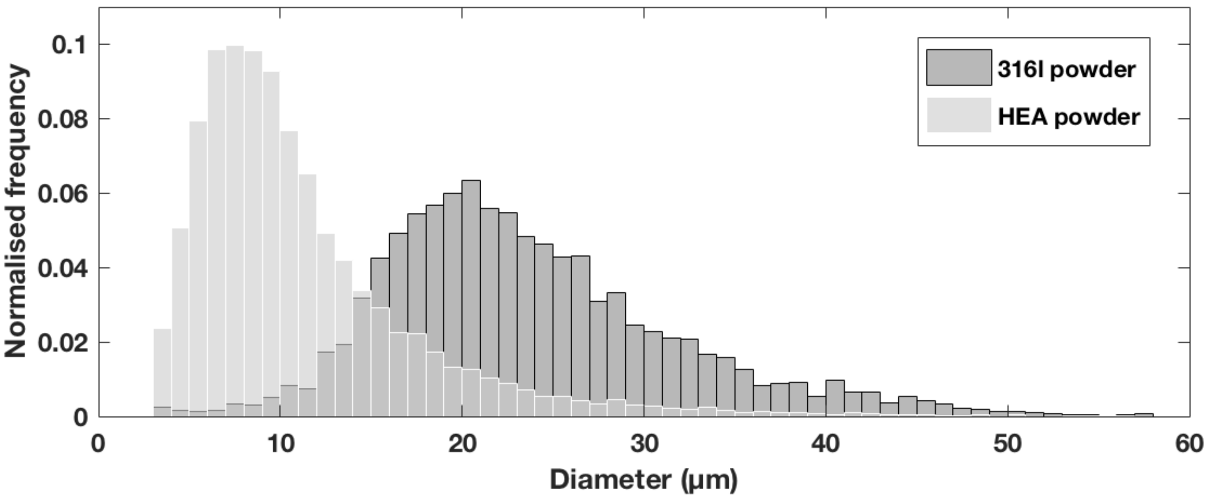

Supplementary Figure 6: Powder size distribution of the 316L and HEA powder. The mean and median of powder size are 22.8 μm and 21.3 μm for 316L steel, and 11.2 μm and 9.5 μm for HEA, respectively.

References:

1. Shan X, Davies CM, Wangsdan T, O'Dowd NP, Nikbin KM. Thermo-mechanical modelling of a single-bead-on-plate weld using the finite element method. *International Journal of Pressure Vessels and Piping* **86**, 110-121 (2009).

2. Hussein A, Hao L, Yan C, Everson R. Finite element simulation of the temperature and stress fields in single layers built without-support in selective laser melting. *Materials & Design (1980-2015)* **52**, 638-647 (2013).

3. Trapp J, Rubenchik AM, Guss G, Matthews MJ. In situ absorptivity measurements of metallic powders during laser powder-bed fusion additive manufacturing. *Applied Materials Today* **9**, 341-349 (2017).

4. Yadroitsev I, Gusarov A, Yadroitsava I, Smurov I. Single track formation in selective laser melting of metal powders. *Journal of Materials Processing Technology* **210**, 1624-1631 (2010).

5. Ding J*, et al.* Thermo-mechanical analysis of Wire and Arc Additive Layer Manufacturing process on large multi-layer parts. *Computational Materials Science* **50**, 3315-3322 (2011).

6. Spierings AB, Levy G. Comparison of density of stainless steel 316L parts produced with selective laser melting using different powder grades. In: *Proceedings of the Annual International Solid Freeform Fabrication Symposium*). Austin, TX (2009).

7. Elmer JW. The influence of cooling rate on the microstructure of stainless steel alloys.). Massachusetts Institute of Technology (1988).

8. Elmer JW, Allen SM, Eagar TW. Microstructural development during solidification of stainless steel alloys. *Metallurgical Transactions A* **20**, 2117-2131 (1989).

9. Qiu C, Kindi MA, Aladawi AS, Hatmi IA. A comprehensive study on microstructure and tensile behaviour of a selectively laser melted stainless steel. *Scientific Reports* **8**, 7785 (2018).

10. Katayama S, Matsunawa A. Solidification microstructure of laser welded stainless steels. *International Congress on Applications of Lasers & Electro-Optics* **1984**, 60-67 (1984).

11. Hooper PA. Melt pool temperature and cooling rates in laser powder bed fusion. *Additive Manufacturing* **22**, 548-559 (2018).

12. Rappaz M, David SA, Vitek JM, Boatner LA. Analysis of solidification microstructures in Fe-Ni-Cr single-crystal welds. *Metallurgical Transactions A* **21**, 1767-1782 (1990).

13. Dovgyy B, Pham M-S. Epitaxial growth in 316L steel and CoCrFeMnNi high entropy alloy made by powder-bed laser melting. *AIP Conference Proceedings* **1960**, 140008 (2018).
