# Supplementary material for: The role of side-branching in microstructure development in laser powder-bed fusion
Source: Nat Commun. 2020 Feb 6;11:749. doi: 10.1038/s41467-020-14453-3 (PMC7004990; doi:10.1038/s41467-020-14453-3)
Supplement: Supplementary file 3 — Description of Additional Supplementary Files [file 41467_2020_14453_MOESM3_ESM.docx]

**Description of Additional Supplementary Files**

**Title: Supplementary Movie 1:** Melt pool of 316L in a modulated laser beam

**Title: Supplementary Movie 2:** Melt pool of 316L in a continuous laser beam
